# Predictors of Caregiver Burden of Patients with Alzheimer Disease Attending Day-Care Centres

**DOI:** 10.3390/ijerph182010707

**Published:** 2021-10-12

**Authors:** María Gómez-Gallego, Juan Cándido Gómez-Gallego

**Affiliations:** 1 Clinical Neuroscience Research Group, Faculty of Health Sciences, Catholic University of Murcia, 30107 Murcia, Spain; mggallego@ucam.edu; 2 Department of Applied Economics, Faculty of Economic, Espinardo Campus, University of Murcia, 30100 Murcia, Spain

**Keywords:** Alzheimer’s disease, burden, caregivers, neuropsychiatric symptoms, kinship

## Abstract

Nowadays, there are plenty of programs and resources to prevent caregiver burden of patients with Alzheimer’s disease. In spite of that, many caregivers suffer high levels of burden and stress, which leads to an earlier institutionalization of patients. This study aimed to explore the predictors of burden in relative caregivers of patients attending day-care centers and the moderating role of caregiver kinship in these associations. A sample of a hundred and two patient–caregiver dyads was recruited. Burden was measured with a Zarit Burden Interview. Measures of patients’ cognition, insight, depression, behavioral disturbances, functional ability and overall physical health were considered as predictors. We found that apathy, irritability and delusions and, patients’ mobility are the main determinants of caregivers’ burden. The strength of relationship between delusions and irritability was higher in spouse caregivers. Interventions to reduce burden should be adapted to the specific needs of a particular type caregiver.

## 1. Introduction

Nowadays, Alzheimer’s disease (AD) is the major cause of disability and functional dependency in the elderly [1]. Moreover, AD affects not only patients’ health and well-being but also that of their caregivers [2,3]. Caregiver burden involves the physical, economic, social and emotional problems experienced by a caregiver of an impaired patient [4]. In fact, burden is a multidimensional construct with objective and subjective aspects [5]. One of the most used instruments to assess burden is the Zarit Burden Interview [6]. Although this scale was developed to measure subjective burden, its score is a good indicator of objective burden and the risk of patient’s institutionalization [7]. Burdened caregivers have worse quality of life, which could influence on the quality of care [8]. In countries with a high prevalence of Alzheimer’s disease, one strategy to improve patients’ care is the utilization of day-care services. These centers offer stimulation and provide respite for informal caregivers. In some studies, the use of a community-based service has been shown to maintain cognitive function and improve patients’ behavior, reduce caregivers’ burden and delay or prevent institutionalization [9,10,11,12,13]. However, sometimes the living situation, health-related factors and special caregivers’ burden lead to the earlier institutionalization of patients [14,15].

The literature on the determinants of burden points out several caregiver and patient-related factors. They include patients’ cognitive impairment, functional dependency and neuropsychiatric symptoms and caregivers’ sense of coherent, coping strategies [16,17,18]. However, little is known about the clinical predictors of burden of caregivers of day-care center patients. This study aims to determine which clinical factors are the best predictors of burden and if these associations depend on a patient–caregiver relationship in these centers. We explore a wide range of clinical variables. We hypothesized that neuropsychiatric symptoms would be the main predictors of burden and that kin relationship may moderate their effect.

## 2. Methods

### 2.1. Participants

The sample consisted of 102 patient–caregiver dyads (204 participants). Patients were selected from day-care centers in the area of Murcia and were considered for inclusion provided they (1) met the Diagnostic and Statistical Manual of Mental Disorders fourth edition (DSM-IV) criteria for dementia of the Alzheimer’s type [19]; (2) were in the mild to moderate stage of dementia (Global Deterioration Scale [20] stage 4 or 5); and (3) live in the community and (4) had a caregiver who maintained regular contact that could act as an informant. Patients with severe communication problems that could not be interviewed were excluded. Caregivers were selected from the relatives who have a higher responsibility of patient care. All the participants signed the informed consent. The study protocol was approved by the Bioethics Committee of the Catholic University of Murcia. Data were collected during the first semester of 2019.

### 2.2. Assessment and Measures

Interviews of patients and caregivers took place separately in the day-care centers. The instruments of assessment were administered by nurses, psychologists and physicians. Caregivers’ burden was assessed using the Zarit Burden Interview [6]. Patients’ cognition was tested with Mini Mental State Examination (MMSE) [21]. Patients’ depression was measured with a self-report scale, the Geriatric Depression Scale [22]. Neuropsychiatric symptoms were assessed with the 12-item Neuropsychiatric Inventory (NPI) [23]. Basic activities of daily living (ADL) were assessed using Barthel Index (BI) [24]. Although BI is used as a unidimensional instrument, factor analysis has pointed out the existence of the following two factors: mobility (transfer, walking and stairs) and personal care (bowels continent, urine continent, grooming, feeding, toilet use, dressing and bathing) [25]. In this study, the scores of both BI factors were considered as predictors. Instrumental ADL was tested with the Functional Activities Questionnaire (FAQ) [26]. Patients’ anosognosia was assessed with Clinical Insight Rating Scale (CIR) [27] and their comorbidity with Cumulative Illness Rating Scale (CIRS) [28].

### 2.3. Statistical Analysis

All analyses were performed using the SPSS version 22. The association analyses between categorical demographic variables and burden were performed using ANOVA. Correlations were calculated between burden scores and both participants’ age and scores of clinical variables, the Spearman’s R was used as we knew the NPI scores were not normally distributed [29]. Possible predictors of burden were selected from those variables that were significant in the bivariant analyses. These variables were introduced in multiple regression models using the default enter method. Collinearity was controlled by means of condition index, proportion of variance and variance inflation factor (VIF).

In order to test the moderating effect of the caregiver–patient relationship on the association between burden and its predictors, we performed hierarchical linear regression analyses [30]. In these models, the order of entry of variables was as follows: step 1 (predictor), step 2 (moderator) and step 3 (interaction between predictor and moderator). A statistically significant interaction indicates the existence of a moderating effect. A negative interaction indicates that the influence of the predictor variable on burden is higher in the spouse caregivers than in adult child caregivers. We examined the interaction of the predictors at each level of moderator in the presence of a significant interaction. The moderation model is described below:

*E*[ZBI] = β_0_ + β_1_X + β_2_M + β_3_predictor × type of caregiver

ZBI is normally distributed. Chi-square = 32.471, df. = 26, Sig. = 0.178.

β_0_ is the population average intercept.

X is the predictor variable. It is a continuous variable.

M is the moderating variable. It is a dummy variable for the type of caregiver (0 for spouses and 1 for adult children).

β_3_ provides an estimate of the moderation effect of the type of caregiver on the associations between predictors and burden.

## 3. Results

The sociodemographic and clinical characteristics of the sample are shown in Table 1. Most of the patients were women, 61 had GDS four and the others had GDS five. Caregivers were moderately overburdened, as the mean ZBI score was 30.66 (range 7–69).

The results of the association analysis between burden and participants’ characteristics are presented in Table 2. There were no significant differences in the ZBI scores with regard to the patients’ gender and marital status. Adult child caregivers had significantly higher ZBI scores than spouses. There were no significant differences in the ZBI scores between female caregivers and male caregivers. The ZBI scores did not correlate with the patients’ years of education and with both the patients’ and caregivers’ age. Significant correlations were observed between burden and the scores of certain NPI symptoms (delusions, hallucinations, agitation, apathy and irritability) and factor mobility of BI.

Firstly, a multiple regression model was estimated considering as predictors all the significant variables in the bivariate analyses. This model was significant (adjusted R^2^ = 32.2; *p* = 0.000) but collinearity was detected among the predictors (Table 3, model 1). There were two variables with variance explained in high proportion in one dimension. Non-significant variables were sequentially removed. Finally, we obtained a model explaining 32.7% of the ZBI variance (Table 3, model 2). All the predictors were significant. The highest condition index was 11.37, which shows that there is no harmful *collinearity* in the model.

The moderating effect of the patient–caregiver relationship was studied separately for the association of burden and each significant predictor. As Table 4 model 1 shows, the type of caregiver (adult child versus spouse) moderates the effect of delusions on the burden. Moderation was significant for the two levels of the moderator (spouse: t = 3.12, *p* = 0.000; adult child: t = 1.36, *p* = 0.080). Likewise, it was observed that the interaction between the type of caregiver and irritability significantly predicted the burden (Table 4, model 2). This effect remained significant for the two levels of the moderator (spouse: t = 3.29, *p* = 0.000; adult child: t = 1.64, *p* = 0.050). The type of caregiver did not moderate the association between burden and both apathy and mobility (*p* > 0.100).

## 4. Discussion

Our findings support the hypothesis of the importance of behavioral symptoms as predictors of burden, even for caregivers who use community-based services. As other studies state, the demographics of participants did not have a notable impact on burden [31]. We cannot rule out an indirect effect of caregivers’ age on burden, due partially to more health problems, less financial resources or different coping strategies in the older caregivers [32,33,34]. The effect of caregiver’s age on burden could be masked by the patient–caregiver relationship. Indeed, we observed that adult child caregivers suffered higher burden than spouses did, which is consistent with other studies [35,36]. One possible explanation could be that adult children usually have other family duties and need to deal with professional problems. Caring for an elderly dependent relative causes a disruption in their family lifestyle and routines [36]. In this study, we explored the moderating effect of the patient–caregiver relationship on the burden.

Among the clinical predictors of burden, global cognitive impairment was not found to be significant. Although some studies have a reported association between measures of cognitive function and burden, the effect of cognition did not remain significant when behavioral disorders are considered in the analyses [36,37]. The literature shows inconsistent findings for the effect of functional disability on burden. Some studies pointed out that the degree of dependence correlated negatively with burden [38,39], and this correlation is higher when both measures were reported by caregivers [35,36,37,38]. The perception of burden may influence the ratings about patients’ functional status [40]. Nevertheless, other studies have concluded that the effect of physical dependence on burden is small. This paradoxical finding may be due to the fact that physical decline is usually accompanied with major provision of care [41]. Furthermore, helping patients to perform some daily living activities has shown to have positive effects on the caregiver, as feeling useful, self-assured, prideful in their abilities to deal with problems and closer to their loved one [42,43].

An interesting result of this study is that caring for patients with better mobility produced higher burden than caring for non-mobile patients. Perhaps, the management of behavioral disturbances requires higher physical effort when patients have good mobility [44,45]. From these disturbances, psychotic symptoms (delusions and hallucinations), agitation, irritability and apathy were found to be the main predictors of burden. The importance of delusions and hallucinations as a source of burden has been recognized in previous studies [44,45,46,47]. These symptoms reveal a higher dysfunction of the frontal lobe and are difficult to control with non-pharmacological interventions, which causes emotional exhaustion [48]. This is also the case of agitation, irritability, disinhibition and executive dysfunction [49]. Apathy is the most frequent and persistent symptom in AD patients and the results are very disturbing to caregivers [41,50,51,52]. In fact, apathy deteriorates the relationship between the patient and the caregiver since it diminishes joint activities and communication [50]. A higher level of effort is necessary to motivate apathetic patients who may rarely even acknowledge the intervention of their caregivers.

As a result, the caregivers’ perceptions of mutuality declines and this may have an influence on subjective burden [52]. Delusions and irritability have a higher effect on spouse caregivers. This can be because spouses are older and have stronger emotional bonds with the patients than adult children; their everyday life is structured around caring for the patients [53]. Despite the efforts to teach caregivers skills to manage neuropsychiatric symptoms, these disturbances are still producing a higher burden. Our results suggest that interventions for preventing and reducing the burden associated to neuropsychiatric symptoms would be more effective when they are focused on treating apathy, irritability and delusions. Concerning apathy, it seems to be useful to implement art therapy programs and other interventions aimed at facilitating patients’ participation in leisure activities that are engaging and stimulating for them [54,55]. Delusions and irritability require a proper pharmacological treatment [56] and also non-pharmacological treatment [57]. According to our results, psychoeducational interventions that provide assistance with psychotic symptom management should be offered, especially to spouses, because they are more vulnerable to burden when these symptoms are present.

A limitation of this research lies in the fact that factors such as personality, mental health/resilience or the quality of the caregivers’ relationship with the patients among others has not been taken into consideration. This limitation could be overcome in future research by reformulating the research goals.

## Figures and Tables

**Table 1:** Demographic and clinical characteristics of the sample.

			%	Mean	SD
Patient *n* = 102	Age			78.38	6.87
	Years of education			4.52	2.69
	Gender	Men	29.8		
		Women	70.2		
	Marital status	Married	59.6		
		Widow/er	40.4		
	MMSE			18.81	6.07
	GDS-15			4.94	3.40
	CIRS			11.36	4.77
	CIR			4.23	2.71
	BI			73.72	19.16
		Factor Mobility		40.96	10.76
		Factor Personal care		32.76	11.11
	FAQ			20.94	8.52
	NPI			30.32	18.47
		Delusions	49.0	2.32	3.43
		Hallucinations	23.5	1.17	2.39
		Agitation	41.2	2.45	3.61
		Depression	64.7	4.62	4.27
		Anxiety	68.6	3.68	3.38
		Euphoria	39.2	2.36	3.35
		Apathy	72.5	5.36	4.25
		Disinhibition	54.9	2.74	3.45
		Irritability	76.5	3.96	3.35
		AMB	47.1	2.74	3.60
		Sleep disorders	45.1	2.60	3.75
		Appetite disorders	39.2	2.79	4.13
Caregivers *n* = 102	Age			59.77	15.57
	Gender	Men		31.9	
		Women		68.1	
	Relationship	Spouse	44.7		
		Adult child	55.3		
	ZBI			30.66	13.55

SD, Standard deviation; MMSE, Mini Mental State Examination; GDS-15, Geriatric Depression Scale; CIR, Clinical Insight Rating Scale; CIRS, Cumulative Illness Rating Scale; BI, Barthel Index; FAQ, Functional Assessment Questionnaire; NPI, Neuropsychiatric.

**Table 2:** Association analyses between patients and caregivers’ factors and burden.

			Mean	SD	F	R
Patient	Age					−0.117
	Gender	Men	32.57	12.90	2.619	
		Women	27.75	16.07		
	Marital status	Married	29.27	13.92	2.184	
		Widow/er	33.40	14.07		
	Years education					−0.101
	MMSE					−0.167
	GDS-15					0.029
	CIR					0.048
	CIRS					0.016
	BI					0.105
		Mobility				0.211 *
		Personal care				−0.023
	FAQ					0.041
	NPI					
		Delusions				0.505 ***
		Hallucinations				0.308 **
		Agitation				0.339 **
		Depression				0.098
		Anxiety				0.110
		Euphoria				0.113
		Apathy				0.238 *
		Disinhibition				0.140
		Irritability				0.289 **
		AMB				0.145
		Sleep disorders				0.088
		Appetite disorders				−0.080
Caregiver	Gender	Men	32.13		0.246	
		Women	30.61	14.84		
	Relationship	Adult child	33.19	13.71	4.205 *	
		Spouse	27.52	12.81		
	Age					−0.145

SD, Standard deviation; F, ANOVA F test; R, Pearson’s correlation coefficient; * *p* < 0.05; ** *p* < 0.01; *** *p* < 0.001; MMSE, Mini Mental State Examination; GDS-15, Geriatric Depression Scale; CIR, Clinical Insight Rating Scale; CIRS, Cumulative Illness Rating Scale; BI, Barthel Index; FAQ, Functional Assessment Questionnaire; NPI, Neuropsychiatric Inventory; AMB, Aberrant Motor Behavior.

**Table 3:** Multiple regression lineal models for predicting burden.

	B	Std Error	β	*p*-Value	Tolerance	VIF
Model 1						
Constant	12,054	5523		0.032		
Delusions	1444	0.447	0.366	0.002	0.568	1762
Irritability	0.715	0.387	0.177	0.068	0.798	1253
Apathy	0.654	0.301	0.205	0.032	0.822	1216
Hallucinations	0.591	0.532	0.104	0.270	0.824	1214
Agitation	0.133	0.461	0.035	0.774	0.485	2062
Mobility	0.221	0.124	0.175	0.078	0.757	1321
Model 2						
Constant	12,528	5197		0.018		
Delusions	1650	0.349	0.418	0.000	0.923	1083
Irritability	0.764	0.353	0.189	0.033	0.954	1048
Apathy	0.752	0.281	0.236	0.009	0.931	1074
Mobility	0.207	0.113	0.164	0.070	0.900	1111

B, Unstandardized coefficient; Std error, Standard error; β, Standardized coefficient, VIF, Variance inflation factor.

**Table 4:** Hierarchical regression analysis testing moderating effects of the type of caregiver on the association between neuropsychiatric symptoms and burden.

	B	Std Error	β	*p*-Value
Model 1				
Constant	30,002	1885		0.000
Type of caregiver	2158	2496	0.080	0.390
Delusions	2937	0.691	0.744	0.000
Delusions*type of caregiver	−1418	0.710	−0.301	0.023
Model 2				
Constant	27,676	1952		0.000
Caregiver	5481	2625	0.202	0.040
Irritability	2045	0.620	0.505	0.001
Irritability*type of caregiver	−1485	0.745	−0.284	0.046

B, Unstandardized coefficient; Std error, Standard error; β, Standardized coefficient. Delusions*type of caregiver is a multiplicative variable where * denotes multiplication.

## Data Availability

Data are contained within the article.
